# An Investigation on the Correlation between the Mechanical Properties of Human Skull Bone, Its Geometry, Microarchitectural Properties, and Water Content

**DOI:** 10.1155/2019/6515797

**Published:** 2019-05-23

**Authors:** Jik Hang Clifford Lee, Benjamin Ondruschka, Lisa Falland-Cheung, Mario Scholze, Niels Hammer, Darryl Chan Tong, John Neil Waddell

**Affiliations:** ^1^Faculty of Dentistry, University of Otago, Dunedin, New Zealand; ^2^Institute of Legal Medicine, University of Leipzig, Leipzig, Germany; ^3^Sir John Walsh Research Institute, Faculty of Dentistry, University of Otago, Dunedin, New Zealand; ^4^Clinical Anatomy Research Group, Department of Anatomy, University of Otago, Dunedin, New Zealand; ^5^Institute of Materials Science and Engineering, Chemnitz University of Technology, Chemnitz, Germany; ^6^Department of Orthopedic, Trauma and Reconstructive Surgery, University of Leipzig, Leipzig, Germany; ^7^Fraunhofer IWU, Dresden, Germany; ^8^Department of Oral Diagnostic and Surgical Sciences, Faculty of Dentistry, University of Otago, Dunedin, New Zealand; ^9^Department of Oral Rehabilitation, Faculty of Dentistry, University of Otago, Dunedin, New Zealand

## Abstract

With increasingly detailed imaging and mechanical analysis, modalities need arises to update methodology and assessment criteria for skull bone analysis to understand how bone microarchitecture and the presence of attached tissues may affect the response to mechanical load. The main aim was to analyze the effect of macroscopic and microstructural features, as well as periosteal attachment, on the mechanical properties of human skull bone. Fifty-six skull specimens from ethanol-phenoxyethanol-embalmed cadavers were prepared from two human cadavers. Assuming symmetry of the skull, all samples from one-half each were stripped of periosteum and dura mater, while the soft tissues were kept intact on the remaining samples on the contralateral side. The specimens were analyzed using microcomputed tomography to assess trabecular connectivity density, total surface area, and volume ratio. The specimens were loaded under three-point bend tests until fracture with optical co-registration. The bone fragments were then lyophilized to measure their water content. With increasingly detailed imaging and mechanical analysis modalities, there is a need to update methodology and assessment criteria for skull bone analysis to understand how the bone microarchitecture and the presence of attached tissues may affect the response to mechanical load. The mechanical properties were negatively correlated to bone thickness and water content. Conversely, most microarchitectural features did not influence either mechanical parameter. The correlation between mechanical response data and morphologic properties remains similar between the results of embalmed tissues presented here and fresh osseous tissue from literature data. The findings presented here add to the existing methodology to assess human skull for research purposes. The interaction between most microarchitectural features in ethanol-phenoxyethanol-embalmed embalmed skull samples and bending stress appear to be minute.

## 1. Introduction

The interest in the mechanical properties of human skull bone grew in the 1960s with the advent of high-speed transportation, focusing on measurement properties like hardness and tensile, shear, and compressive strength [1–4]. Early studies from the 1970s to first address this topic did not account for the complexity of stresses that transmit through the irregularly shaped skull. Later studies showed a shift in paradigm towards investigation of further load responses such as elastic modulus (ranging between 2.0 and 18.1 GPa) and bending strength (ranging between 64.3 and 133.6 MPa), some of which are displayed in Supplemental Table 1 [5–9].

Further investigation into structural properties of skull bones with increasingly advanced technology revealed more factors that may potentially affect mechanical response. In particular, development of computational (finite element) models has generated an area of study to assess the effect of individual properties such as sampling sites, geometry, and tissue condition of different skull bone samples [7, 8, 10].

Skull bone thickness and density are the main factors that influence the mechanical properties of skull bone: specifically, a strong positive correlation between skull bone thickness and stiffness is most frequently observed [7, 8]. The volume and mass of skull bone is another modulator for the mechanical response of skull bone to load. Auperrin et al. [7] observed that increased density was correlated with an increase in elastic modulus. Motherway et al. [6] described similar trends with positive correlations between percentage bone volume, and bending modulus and bending strength.

A lateral symmetry in mechanical properties was described for skull bones [7, 8], with the highest elastic moduli observed in temporal bones (5.2–6.0 GPa), to parietal bones (3.8–4.5 GPa), and then the frontal bone (3.3 GPa). There were no studies that specifically investigated properties of the occipital bone despite its crucial protective role, considering the frequency of trauma to the back of the head. Only a brief reference ascribed greater thickness to higher fracture loads in occipital bones [9].

Studies involving animal tissues have observed that water (or fat) content in bones can influence the mineral density of bone, thereby affecting their strength [11]. Specifically, there is a significant negative correlation observed between water content and bending modulus of bone [12, 13].

Microcomputed tomography (*µ*-CT) scanning has increased the sensitivity of bone density measurements and opened up quantitative measurement of new microarchitectural parameters. Early computational finite element models observed limited functional correlation between connectivity density (defined as the three-dimensional trabecular connections, being the number of linking elements between the trabeculae over a given volume) and elastic modulus [14], but more recent *in vivo* analyses of cancellous bone determined that certain architectural parameters such as trabecular connectivity and overall bone density distribution can be strong determinants of mechanical response [15, 16].

This given work aimed to measure the mechanical response of human skull bones to a static load and to assess the correlation with its geometric and microstructural properties. An image correlation technique for displacement measurement as described elsewhere [17] was used to measure mechanical properties, and the effect of variables such as geometry, water content, bone volume fraction, bone microarchitecture, and the presence of periosteum and dura mater on mechanical response was assessed.

## 2. Materials and Methods

### 2.1. Subjects

Two chemically fixed cadavers, one male aged 61 and one female aged 86, were taken as subjects. These tissues were embalmed in a mixture of ethanol, glycerin, formaldehyde, and phenoxyethanol at effective concentrations of 12.7%, 3.4%, 1.5%, and 0.4%, respectively. Tissues were selected following gross anatomical inspection of soft and hard tissues of the head and using clinical imaging datasets available for the cadavers. Exclusion criteria for selecting the cadavers included pathology that may affect bone properties (e.g., osteoporosis and Paget's disease) and vital trauma to the head that may have damaged the skull.

Ethical approval was granted by the University of Otago Human Ethics Committee (Health) (ref: H17/02) prior to specimen collection. Māori consultation for the project was sought from the Ngāi Tahu Research Consultation Committee.

### 2.2. Samples

**Figure 1:**
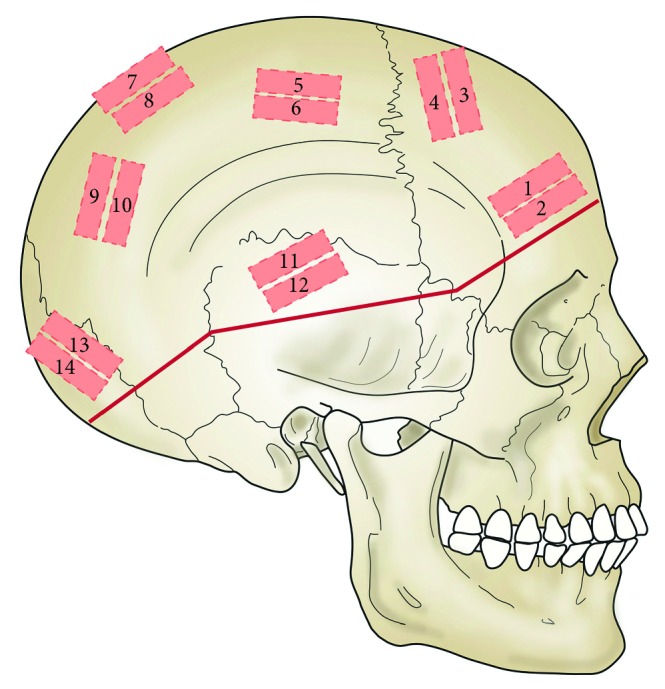
Diagram of sampling sites of specimens mapped on a skull.

Twenty-eight specimens were prepared from each calvarium (total *n*=56) according to a specific map of the skull (Figure 1). High flow water irrigation was used during cutting to prevent overheating of the tissues.

Sectioned specimens were further refined through hand sanding under irrigation with metallographic grinding paper at grit size 162 *µ*m (P100), followed by 82 *µ*m (P180), until it reached the final dimension of 10 × 40 mm (±0.25 mm) as measured by a digital micrometer.

Assuming symmetry of the skull, the specimens from one randomly chosen side including all the subsamples were stripped of periosteum and dura mater as a control group (*n*=14 per skull), and the periosteum and dura mater were kept intact on the other side (*n*=14 per skull). This resulted in a full sample size consisting of 56 bone specimens, of which 28 had attached soft tissues distributed over two skulls. The prepared skull bone specimens were stored at room temperature in a 2% phenoxyethanol preservative solution until required and thoroughly rinsed in isotonic saline prior to the mechanical tests.

### 2.3. Mechanical Tests and Image Analysis

**Figure 2:**
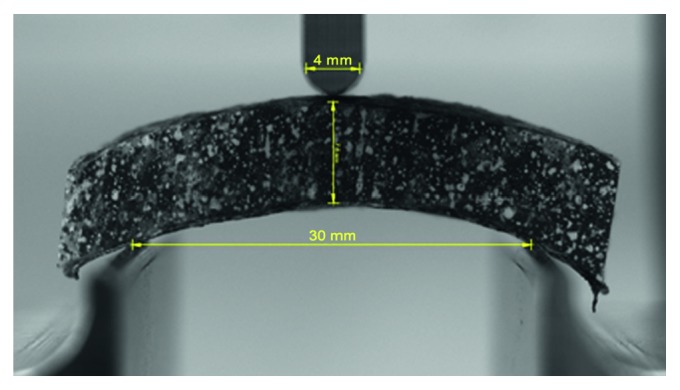
A skull specimen on the three-point bending rig, with loading beam radius 2 mm and support beams 30 mm apart. Measurements were taken from the mid distance of the diploë layers at the horizontal orientation of the upper and the lower anvils.

Prior to the mechanical testing, tissue specimens were rehydrated with isotonic 0.9 mass% saline for at least 12 hours to reduce the effects of desiccation. Specimens were loaded onto a three-point bend testing rig on a universal testing machine (Z020, Zwick Roell Group, Ulm, Germany) on support beams with beam radii of 1 mm set 30 mm apart. On the loading arm, a plunger with tip radius of 2 mm was used (for visualization, see Figure 2). Specimens were loaded at 10 mm·min^−1^ until fracture.

A digital image correlation system (Limess Messtechnik und Software GmbH, Krefeld, Germany) was used synchronized with the testing machine to capture test footage. Proprietary software (Istra4D, Dantec Dynamics, Skovlunde, Denmark) was used to identify regions of interest on the front and back of each specimen, and topographic maps of strain distribution as well as overall vertical displacement were determined. This was used to measure the deflection at the base of the specimen in the *Y* (vertical) axis, at the lowest point of the sample under the loading plunger at both sides. Bending stress and bending strain were computed as follows, assuming a flexural deformation of a bending beam (rectangular cross section) with the average dimensions measured. The final properties of each specimen as a whole were calculated from the average of values from the front and back camera datasets:

(1) $$\begin{array}{c} \text{bending stress}=\frac{3\;\times \text{ force }\left(\text{N}\right)\;\times \text{ span }\left(\text{mm}\right)}{2\;\times \text{ width }\left(\text{mm}\right)\;\times \text{ thickness }{\left(\text{mm}\right)}^{2}},\\ \text{bending strain}=\frac{6\times \text{deflection }Y\;\left(\text{mm}\right)\;\times \text{ thickness}\;\left(\text{mm}\right)}{\text{span }{\left(\text{mm}\right)}^{2}}. \end{array}$$

Bending strength was defined as the maximum of the bending stress-strain curves. Bending modulus was evaluated as the linear slope of each bending stress-strain graph.

### 2.4. Geometric and Microstructural Property Analysis

Gross dimensions including specimen thickness and width of the bone were measured manually with a digital micrometer. The width of each specimen was measured at the midpoint, on a line perpendicular to the longitudinal edge. The average thickness of each specimen was calculated with measurements along the midline.

A Skyscan 1172 *µ*-CT scanner (Bruker, Belgium) was used to accurately measure microarchitectural properties of all skull specimens using a similar approach outlined elsewhere [18]. A custom specimen holder was 3D printed with a radiolucent polylactic acid thermoplastic (Ultimaker 3, Ultimaker, Geldermalsen, The Netherlands) in preparation for scanning. The specimens were scanned with settings at medium resolution with image pixel size 17.45 *µ*m, rotation step 0.5°, source voltage 100 kV, and source current 100 *µ*A. The volume of the scan was 40 mm (specimen length) × 30 mm (radius of the sample holder for six samples). Using the ImageJ (version 1.51k, National Institutes of Health, MD, USA) and BoneJ (version 1.4.2, London, UK), the specimens were segmented and processed with a specialized plugin to measure properties of connectivity density, total bone surface area (surface area of all trabecular and cortical layers within the bone), and trabecular volume ratio (defined as trabecular volume divided by bone volume).

Following the mechanical tests, the specimens were weighed prior to and following freeze-drying to determine the percentage of water by weight (%wt.) according to the procedures shown elsewhere [19]. In brief, the tissue weight was determined prior to freezing and exposure to vacuum for water removal and then reweighed afterward.

### 2.5. Statistical Analysis

For statistical analysis, PRISM 7 software (GraphPad, CA, USA) was used. Each skull was individually analyzed. Normality was determined using the Kolmogorov–Smirnov test. Wilcoxon matched-pairs tests (nonparametric) were used to compare bare and periosteum-attached bone specimens of each skull individually. Furthermore, Spearman's correlation tests (nonparametric) were used to identify correlations between the bending modulus and bending strength, and independent variables measured (i.e., thickness, water content, connectivity density, total bone surface area, and trabecular volume ratio).

## 3. Results

The mean values and standard deviations of the independent variables measured are listed in Table 1.

The mean thicknesses of specimens were not statistically different between bare bone and samples with attached soft tissues comparing all data (*p*=0.084). Water content was significantly higher in the bare bone skull samples (*p*=0.013 for skull 1 and *p*=0.001 for skull 2).

The mechanical properties of the skull specimens are described in Table 2, indicating the difference between bare bone samples and specimen with attached soft tissues with statistical significances. There were significant differences in all properties except in skull 1 for bending strength.

**Figure 3:**
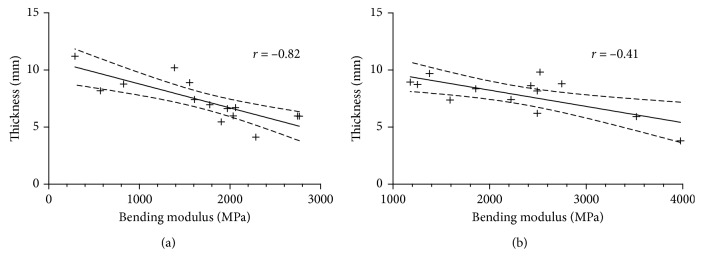
Scattered plots and corresponding regression lines with 95% confidence intervals depicting the association of specimen thickness and bending modulus (see Table 3 for *p* values). (a) Specimen 1; (b) specimen 2.

To rule out contributions to these differences, the correlation between mechanical and independent properties such as specimen thickness, water content, connectivity density, total surface area, and trabecular volume ratio was calculated and tabulated in Table 3. Scattered plots for the correlation of thickness and bending modulus are illustrated in Figure 3.

**Figure 4:**
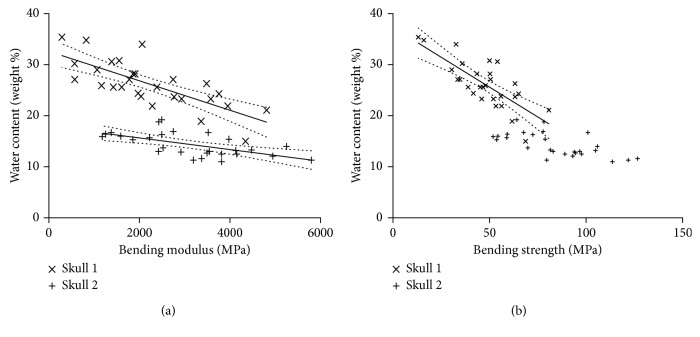
Scattered plots and corresponding regression lines with 95% confidence intervals depicting the association of bending modulus (a) and bending strength (b) with the water content of the bone specimens (see Table 3 for *p* values).

**Figure 5:**
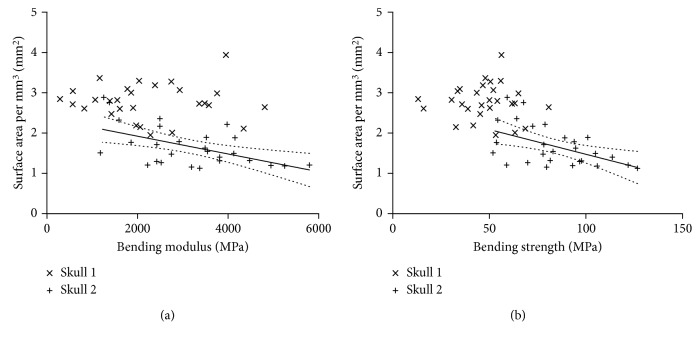
Scattered plots and corresponding regression lines with 95% confidence intervals depicting the association of bending modulus (a) and bending strength (b) with the surface area of the bone specimens (see Table 3 for *p* values).

There were significant negative correlations between mechanical response and macroscopic parameters such as thickness and water content (see Figure 4) and to total surface area with significant results in skull 2 (Figure 5). However, microarchitectural features did not have many significant correlations in total. The only positive significant correlation was found for bending modulus and trabecular volume ratio in skull 2.

## 4. Discussion

This project aimed to develop a protocol to measure the geometric and microarchitectural properties of human skull bones and to assess its correlation with the mechanical response in the form of bending modulus and bending strength in three-point bending. A significant influence of attached periosteum and dura mater on both bending modulus and bending strength of human skull samples was demonstrated in this study. Furthermore, mechanical response was negatively correlated to the bone specimen thickness and water content. No significant correlations were observed between microarchitectural features and mechanical response. The first observation is that the values for bending modulus fell within formerly reported ranges [7, 8] and in skull 1's periosteum-attached and skull 2's bare specimens, the bending strength coincided directly with values reported by Torimitsu et al. [9]. The values observed in this study were comparably lower than other reported means, possibly due to the difference in sample size, age at death, and the effects related to the embalming of the tissues. The influence of chemicals used as fixatives appears to be dependent on the type and origin of tissue as well as the concentration and the duration of the chemicals being used. It is well known that chemical embalming processes involving such chemicals may cause embrittlement of tissues. Controversial results exist for formaldehyde on bone tissues [20, 21], while the effects of ethanol appear to differ regarding their reversibility [21]. However, the thorough rinsing protocol with isotonic saline prior to specimen collection and testing has been proven to return elastic modulus of tissues to, or below, prefixation values in bones and ligamentous tissues [22, 23].

Investigators of other studies cited here had access to larger numbers of fresh tissues, obtaining primarily baseline mechanical properties. Their approach, consequently, aimed at answering different research questions from the one addressed here. In comparison, this investigation was carried out on embalmed skull samples with the aim of establishing a protocol to accurately compare mechanical and microarchitectural properties for cranial bone samples. A recent study conducted by Alexander and coworkers [18] utilized *μ*-CT in frontal and parietal bones of cadaveric heads and analyzed the substructures of the skull. They observed site-dependent properties and found random distribution patterns of the diploë in the transverse plane in a sample of four cadavers [18]. In spite of the relatively small number of samples, the authors have been able to utilize their data for modelling purposes.

Equally, our study would also strongly benefit from a greater sample size and use of fresh tissues, i.e., the number of cadavers was limited to two, and the underlying individual morphology may have influenced our numerical results. However, we primarily aimed at determining structural-mechanical relationships. Our results should consequently be a starting point for comparison to fresh skull bone behavior, and further tests with fresh tissues will create a basis to compare results to that of different skull simulants for reconstruction purposes [24], which our research group is already working on. A surprising observation was that comparison of the sample thicknesses between left and right, i.e., bare bone vs. bone with periosteum, yielded inconclusive differences in specimen thickness. More specifically, bone seemed thinner in one of the samples with periosteum and dura attached compared to the contralateral bare side. These findings may indicate that the assumption of symmetry of skull bone thickness may not be given in humans.

### 4.1. Mechanical and Macroscopic Properties Are Influenced by the Adjacent Soft Tissues

Despite the varied methodologies and tissue variables, it was interesting to note the similarities in results that were present, with nearly exactly comparable significant negative correlations to the mechanical properties investigated [7, 13]. Despite these similarities, however, it is important to note that some referenced studies were conducted on non-human bone tissues [12, 13]. Previous investigations on the mechanical properties of bone have always been conducted with osseous tissues in isolation. This is the first time in reported literature that the periosteum and dura mater of skull specimens have been considered as part of a functional unit during mechanical load. There was a statistically significant difference in bending modulus observed between bare bone and periosteum-attached specimens. Considering the assumption of sagittal symmetry in other macro- and microarchitectural variables [7] and our own statistical comparison, the difference in mechanical properties between these two sample collections indicate that removal of soft tissues in skull bone biomechanics must be attributed as a relevant confounder in data analysis and comparison to real-life scenarios. However, the effect of soft tissues on parameters such as water content must also be accounted for before a definitive conclusion can be made on its effect on mechanical properties.

### 4.2. Microarchitectural Properties Do Not Affect Mechanical Response of Skull Bone Relevantly

The minor influence of bone connectivity density to any mechanical properties is consistent with existing observations of trabecular bone under compressive loads [12, 15, 25, 26]. It was postulated that the lack of correlation is due to bone connectivity density being independent of the nature of the trabeculae, where thickness and geometry (i.e., rods or plates), and thereby strength and rigidity, can vary despite an unchanged number of connections [14]. Unfortunately, to our best knowledge, there was no previous analysis of bone connectivity density under beam loading for comparison to our recent data. This investigation found poor significance and lack of correlation between trabecular volume ratio (proportion of total volume occupied by bone to that by trabecular architecture) and mechanical properties. Conversely, other investigators observed that trabecular volume ratio was a potential predictor of compressive mechanical properties [15, 25–27]. However, it is impossible to make inferences on this basis considering the different manners of mechanical loading used here and elsewhere.

### 4.3. Limitations

The mechanical loading scenario used here was limited to a static approach, though the skull is loaded dynamically especially under traumatic conditions leading to bone injury. Furthermore, the simplification of a straight bending beam with rectangular cross section was used to calculate the bending stress and strain of each sample. This approach was used because of the complexity of sample geometries from straight to different curvatures. Due to the sample size, specimens collected from various sites of the skull (i.e., frontal, parietal, temporal, and occipital bones) were not considered separately but may show regional different biomechanical behavior in larger collections. Next to the small sample size, the authors could not rule out with certainty any influence of the cadavers' age at death and gender on its mechanical properties. Both skulls represent geriatric osseous tissues, and we are aware of age and gender influence at least towards some anatomical landmarks of the skull, allowing for forensic investigation and identification [28, 29]. However, to date, little is known concerning the biomechanical response of skull bones in regard of changing ages and different sexes of tested individuals, but this should be an aim for future studies. Furthermore, it will be of interest to investigate the site-specific properties of human skull bone for the cortical and cancellous layers separately, involving both morphology and mechanical properties.

## 5. Conclusions

This investigation was able to reliably assess skull bone tissues, measuring and comparing relevant biomechanical data with macroscopic and microarchitectural properties. Significant correlations of bone thickness and water content to mechanical response seem to be similar between embalmed and literature-reported fresh osseous skull specimens, validating the protocol used in this study. The presence of attached soft tissues also contributed towards statistically significant differences in mechanical properties. However, most microarchitectural features did not appear to influence bending modulus or bending strength, and there was a lack of comparable data in published literature. Further investigation involving a greater sample size and fresh tissues will be required to validate the statistical findings.

## Figures and Tables

**Table 1:** Different variables of the used bone samples with mean ± standard deviation.

	Skull 1 bare bone	Skull 1 with soft tissues	Skull 2 bare bone	Skull 2 with soft tissues
Thickness (mm)	7.32 ± 1.87	5.75 ± 1.84	7.83 ± 1.63	8.40 ± 1.96
Width (mm)	10.12 ± 0.21	10.08 ± 0.32	10.46 ± 0.23	10.09 ± 0.32
Water content (%wt.)	28.40 ± 4.21	23.97 ± 3.66	16.38 ± 1.37	12.45 ± 0.86
Connectivity density (mm^−3^)	4.11 ± 2.48	5.40 ± 5.42	4.53 ± 2.30	4.78 ± 7.38
Bone surface area (mm^2^ per mm^3^)	2.64 ± 0.42	2.91 ± 0.42	1.96 ± 0.52	1.39 ± 0.23
Trabecular volume ratio (%)	0.71 ± 0.11	0.64 ± 0.11	0.84 ± 0.08	0.89 ± 0.05

**Table 2:** Mean mechanical properties, standard deviation, and mean difference between skull specimens.

	Bare bone	Bone with attached soft tissue	Mean difference
*Skull 1*			
Bending modulus (MPa)	1,699 ± 712	2,282 ± 811	1,065^*∗*^
Bending strength (MPa)	42 ± 14	68 ± 13	11

*Skull 2*			
Bending modulus (MPa)	2,737 ± 1300	3,952 ± 893	1,728^*∗∗*^
Bending strength (MPa)	53 ± 13	99 ± 14	32^*∗*^

^*∗*^ *p* < 0.05; ^*∗∗*^ *p* < 0.001.

**Table 3:** Spearman correlation coefficients assessing relationships between mechanical properties and independent variables.

	Correlation coefficient for independent variables (*r* value)				
	Thickness	Water content	Connectivity density	Total surface area	Trabecular volume ratio
*Skull 1*					
Bending modulus	−0.82^*∗∗*^	−0.78^*∗∗*^	−0.31	−0.08	0.27
Bending strength	−0.60^*∗∗*^	−0.71^*∗∗*^	−0.015	−0.02	0.15

*Skull 2*					
Bending modulus	−0.41^*∗*^	−0.59^*∗*^	−0.26	−0.43^*∗*^	0.41^*∗*^
Bending strength	0.03	−0.65^*∗∗*^	−0.35	−0.48^*∗*^	0.30

^*∗*^ *p* < 0.05; ^*∗∗*^ *p* < 0.001.

## Data Availability

The full data used to support the findings of this study are available from the corresponding author upon request.

## Supplementary Materials

Supplementary Materials Supplemental Table 1. Different properties of skull bone reported in more recent literature.
