# Supplementary material for: An Investigation on the Correlation between the Mechanical Properties of Human Skull Bone, Its Geometry, Microarchitectural Properties, and Water Content
Source: J Healthc Eng. 2019 May 23;2019:6515797. doi: 10.1155/2019/6515797 (PMC6556309; doi:10.1155/2019/6515797)
Supplement: Supplementary Materials — Supplemental Table 1. Different properties of skull bone reported in more recent literature. [file 6515797.f1.pdf]

**Supplemental Table 1.**Different properties of skull bone reported in more recent literature.

| | **Delille et al. (2007) [5]** | **Motherway et al. (2009) [6]** | **Auperrin et al. (2014) [7]** | **Rahmoun et al. (2014) [8]** | **Torimitsu et al. (2015) [9]** |
|---|---|---|---|---|---|
| **Testing scenario** | Three-point bending | Three-point bending | Three-point bending | Three-point bending | Three-point bending |
| **Velocity** | 10 mm/min | 0.5, 1, 2.5 m/s | 10 mm/min | 10 mm/min | 6 mm/min |
| **Properties** | | | | | |
| **Elastic modulus (in GPa)** | 3.6-11.3 | 4.4-18.1 | 3.8-9.8 | 2.0-6.0 | *Not reported* |
| **Bending strength (in MPa)** | *Not reported* | 78.2-133.6 | *Not reported* | *Not reported* | 64.3-85.8 |
| **Stiffness (in N/mm)** | *Not reported* | *Not reported* | *Not reported* | 265-640 | *Not reported* |
